# Impact of schistosome infection on long-term HIV/AIDS outcomes

**DOI:** 10.1371/journal.pntd.0006613

**Published:** 2018-07-02

**Authors:** Soledad Colombe, Richard Machemba, Baltazar Mtenga, Peter Lutonja, Samuel E. Kalluvya, Claudia J. de Dood, Pytsje T. Hoekstra, Govert J. van Dam, Paul L. A. M. Corstjens, Mark Urassa, John M. Changalucha, Jim Todd, Jennifer A. Downs

**Affiliations:** 1 Center for Global Health, Weill Cornell Medicine, New York, New York, United States of America; 2 National Institute of Medical Research, Mwanza, Tanzania; 3 Department of Medicine, Bugando Medical Centre, Mwanza, Tanzania; 4 Department of Molecular Cell Biology, Leiden University Medical Centre, Leiden, Netherlands; 5 Department of Parasitology, Leiden University Medical Centre, Leiden, Netherlands; 6 Department of Applied Biostatistics, London School of Hygiene and Tropical Medicine, London, United Kingdom; University of Washington, UNITED STATES

## Abstract

**Background:**

Africa bears the burden of approximately 70% of global HIV infections and 90% of global schistosome infections. We sought to investigate the impact of schistosome infection at the time of HIV-1 seroconversion on the speed of HIV-1 disease progression, as measured by the outcome CD4+ T-cell (CD4) counts <350 cells/μL and/or death. We hypothesized that people who had been infected with *Schistosoma* spp. at the time they acquired HIV-1 infection would have impaired antiviral immune response, thus leading them to progress twice as fast to a CD4 count less than 350 cells/μL or death than would people who had been free of schistosomes at time of HIV-1 seroconversion.

**Methods and principal findings:**

We conducted a longitudinal study in Tanzania from 2006 to 2017 using stored blood spot samples, demographic surveillance and sero-survey data from the community, and a review of clinical charts. A competing risk analysis was performed to look at the difference in time to reaching CD4 counts < 350 cells/μL and/or death in HIV-1-infected people who were infected versus not infected with *Schistosoma* spp. at time of HIV-1 seroconversion. We found an 82% reduction in risk of reaching the outcome in seroconverters who had been infected with *Schistosoma* (subHazard Ratio = 0.18[0.068,0.50], p = 0.001) after adjusting for age, occupation, clinic attendance and time-dependent covariates.

**Conclusions:**

Our study demonstrates that people with schistosome infection at the time of HIV-seroconversion develop adverse HIV outcomes more slowly than those without. The findings are contrary to our original hypothesis. Our current longitudinal findings suggest complex interactions between HIV-1 and schistosome co-infections that may be modulated over time. We urge new immunological studies to investigate the long-term impact of schistosome infection on HIV-1 viral load and CD4 counts as well as related immunologic pathways.

## Author summary

Several studies had shown that people infected with schistosome parasites were at risk of getting HIV and that they may have higher HIV viral concentrations in their blood as well. The authors explored the impact of the parasite on HIV disease after infection, expecting that people co-infected with the parasite at time of HIV-infection would develop worse HIV outcomes than people who did not have the parasite. HIV seroconverters were identified among a large cohort tested regularly for HIV in northwest Tanzania and were followed-up in clinics to determine their CD4 + T-cells counts and mortality. The seroconverters’ stored blood samples were tested for infection with the parasite and outcomes were compared between seroconverters who were and were not infected with the parasites at the time that they became HIV-infected. The authors surprisingly found that people with the parasite experienced fewer negative outcomes of HIV than people who did not have the parasite. To the investigators’ knowledge, this is the first longitudinal study to find this result. Long-term immunological changes may explain this protective effect and more studies are recommended to explore this research question.

## Introduction

Among approximately 36.7 million global HIV infections, an estimated 6 million individuals are schistosome co-infected [1–3]. Multiple studies have reported interactions between infection with *Schistosoma* spp. and HIV-1 in humans. A recent longitudinal study in Tanzania demonstrated that schistosome infection is a risk factor for HIV-1 acquisition in women, but not in men [3]. These prospective findings substantiated four different cross-sectional studies from Tanzania and Zimbabwe that had shown a ~3-fold increased odds of HIV-1 infection in women with schistosome infections compared to those without, and no increased odds in men [4–7]. Local physical and immunological changes caused by schistosome eggs in the mucosal tissue of the vagina and cervix are thought to increase susceptibility to the virus during sexual HIV exposure [8–10], providing a mechanism for how schistosome infection could increase the risk of incident HIV-1 infection in women. In contrast, men with schistosome infections may not have increased susceptibility to HIV-1 infection because schistosome eggs in men primarily affect internal genital organs, such as the prostate, that are not exposed to HIV-1 during sexual contact [7, 11–12].

In addition to demonstrating increased HIV-1 susceptibility, the longitudinal study in Tanzania also found increased HIV-1 RNA viral load set-points in both men and women who were infected with *Schistosoma* spp. at time of HIV-1 seroconversion [3]. Some, though not all, additional studies have demonstrated that treatment of *Schistosoma mansoni* is associated with a decrease in HIV-1 viral load in co-infected individuals [8,13]. Therefore, it is conceivable that schistosome infection at the time of HIV-1 acquisition increases the HIV-1 viral load set-point, which in turn could accelerate AIDS-related-outcomes [14]. The median increase of 0.7 log_10_ copies/mL that was observed in the study would be predicted to increase the time to AIDS or death by 2 to 3 years [3, 15].

We thus sought to investigate the impact of schistosome infection at the time of HIV-1 seroconversion on the speed of HIV-1 disease progression, as measured by CD4+ T-cell (CD4) counts and mortality. We hypothesized that people who had been infected with schistosomes at the time they acquired HIV-1 infection would have impaired antiviral immune response, thus leading them to progress twice as fast to a CD4 count less than 350 cells/μL or death compared to people who had been free of schistosomes at time of HIV-1 seroconversion.

## Methods

### Study setting and design

#### Identification of HIV-seroconverters

Our study was conducted within the ongoing TAZAMA project, a community-based longitudinal open HIV-testing cohort in Kisesa, northwest Tanzania, which has conducted sero-surveys in a population of ~20,000 adults since 1994. Adults are offered voluntary HIV testing and counseling and provide dried blood spots (DBS) every three years. A Demographic Surveillance System documents population characteristics, births, migration, and deaths every six months. Additional details have been previously described [16–17]. For this study, we identified seroconverters who became HIV-1-seropositive between September 2006 (sero-survey 5) and February 2016 (sero-survey 8), our enrollment period, and who had archived DBS or serum available for testing. Seroconverters were defined as individuals who had been HIV-1-seronegative in one sero-survey and who were found to be HIV-1-seropositive in a following sero-survey. Seroconverters’ demographic data was obtained through linkage to the demographic surveillance data.

#### Follow-up

The follow-up period spanned from date of seroconversion to March 15^th^ 2017. The date of seroconversion was approximated as the mid-point between the last negative DBS and the first positive test, either at a sero-survey or at a clinic. In order to assess the clinical outcomes of HIV-1 seroconverters, we searched for each seroconverter manually and via computer algorithm, by name, sex, date of birth, and place of residence in all the health clinics providing HIV care within a 10 km radius around the sero-survey catchment area. We additionally visited the two oldest and largest HIV clinics in the region (in Mwanza City, 20 km from the demographic surveillance system area) to search for seroconverters. The demographic surveillance data were also used to obtain vital status of HIV-1-seroconverters.

Data about patients seeking care for HIV infection was extracted from both paper files and computer databases. Data that was collected included antiretroviral treatment use and adherence, co-infection with tuberculosis, WHO-defined HIV/AIDS clinical stage, CD4 count, weight, co-infection with other sexually transmitted infections, and comorbidities such as self-reported hypertension and diabetes.

Since 2004, CD4 count monitoring has been used to assess ART eligibility. Until 2010, the criteria for ART initiation were a CD4 count ≤200 cells/mm^3^ or a WHO clinical stage of 4 for all adults. From 2010 to 2012, the criterion was CD4 count ≤350 cells/mm^3^. From 2013 to 2015, it changed to CD4 count ≤500 cells/mm^3^ and finally in 2016 any HIV-positive individual was eligible to initiate ART [18–21].

#### Schistosome infection status

Determination of schistosome infection status was made by measurement of schistosome Circulating Anodic Antigen (CAA) in DBS collected during two successive sero-discordant sero-survey visits. The CAA test is a genus specific assay and thus does not differentiate between *mansoni* and *haematobium* species present in the Kisesa area of Tanzania. Our group has previously shown that approximately 40% of the adults have *S*. *mansoni* infection and 2% have *S*. *haematobium* infection [5,7].

We defined schistosome positivity at time that a person became HIV-1 infected as having a positive test for schistosome infection in that person’s DBS collected both at the last sero-survey where he/she tested negative for HIV-1 and at the first sero-survey where he/she tested positive for HIV-1. If at least one of the two DBS was negative for schistosome infection, the individual was defined as schistosome negative at time of HIV-1 sero-conversion.

### Laboratory testing

#### Dried blood spots

DBS were collected by finger prick with a fingerstick lancet onto a Whatman Protein Saver 903 card (GE Healthcare Bio-Sciences, Pittsburgh, PA). Each spot of blood is 13 millimeters in diameter. DBS cards were dried out of direct sunlight and sealed in a gas-impermeable zip bag with desiccant and humidity indicator. Cards were stored at the NIMR laboratory in Mwanza at -20°C.

#### HIV-1 testing

Diagnosis of HIV-1 infection was confirmed by two different tests in accordance with current national HIV guidelines at each time point. In sero-surveys 5 and 6, the Uniform II Category III Ab test was used as the screening test and the Enzygnost test was used as the confirmatory test. In sero-survey 7, the Uniform II Category IV Ab+Ag test was used as the screening test and Enzygnost was used as confirmatory test. In sero-survey 8, the Determine test was used as the screening test and the Unigold test was used as the confirmatory test. For sero-surveys 5 to 7, DBS were tested at the NIMR laboratory. For sero-survey 8, rapid tests were used on site for screening and confirmation of HIV-1, and around 10% of the stored DBS were tested at NIMR as quality check for the rapid test results. If the sample was negative at the screening test the final result was reported as negative. A sample that was positive at the screening test was tested with the confirmatory test. If the confirmatory test was negative, the final result was reported as negative. If the confirmatory test was positive, the final result was reported as positive. At the Bugando Medical Centre clinical laboratory in Mwanza, Tanzania, CD4 counts were measured using an automated BD FACS Calibur Machine (BD Biosciences, San Jose, CA, USA).

#### *Schistosoma* spp. testing

DBS were tested for schistosome CAA at Leiden University Medical Center by eluting whole blood from DBS and then concentrating the sample as previously described [22], with minor modifications. A total of 226 mm^2^ of DBS were placed into 500μL of phosphate-buffered saline and incubated for 30 minutes at room temperature and then overnight at 4°C. The next day, samples were placed on a shaker for 30 min at room temperature and 30 min at 37°C, after which 250μL of 6% (w/v) tricholoroacetic acid (TCA) was added. The mixture was vortexed, centrifuged, and concentrated using an Amicon 0.5 mL concentration device (Merck, Darmstadt, Germany). The concentrate was then used in the standard CAA UCP assay.

A lower limit threshold of 2 pg CAA per mL of eluted blood was used for the assay. Eleven individuals had stored serum samples but no DBS samples available for testing and underwent serum CAA testing at NIMR with a lower limit threshold of 30 pg CAA per mL [23]. Samples scoring values above the threshold were designated positive for *Schistosoma* infection.

### Statistical analysis

Analysis included all individuals who HIV-1 seroconverted between September 2006 and February 2016. Binary variables were described as proportions and continuous variables were described using median and interquartile range. We assessed differences in baseline clinical characteristics between schistosome positive seroconverters and schistosome negative seroconverters using Chi-square or Fisher’s exact test for proportions and the nonparametric equality test for medians.

A competing risk analysis was conducted to look at the difference in time to outcome in HIV-1 seroconverters who were infected versus not infected with *Schistosoma* at time of HIV-1 seroconversion. The outcome of interest was defined as a composite endpoint: either CD4 count <350cells/μL or death. The competing risk event was defined as start of antiretroviral treatment (ART) when occurring without a preceding outcome, since after ART initiation the risk of reaching the outcome of interest becomes very small. Data was censored for loss to follow-up, defined as the latest of 3 months after the last clinic visit or 1 year after the last demographic surveillance visit. All study participants were censored on 15^th^ March 2017 for this analysis. The cumulative incidence function method was used to assess and compare time to outcome between schistosome infection groups. A competing risk regression with subdistribution hazard analysis, adjusted for all significantly different baseline factors as well as biologically sound variables, was used to assess endpoint incidence difference by schistosome infection status while controlling for ART initiation. Variables that were associated with the outcome at a 10% significance level were individually included into a step-wise analysis and model goodness-of-fit assessed.

Using the methods of Latouche et al. [24] to calculate sample size for subdistribution hazard ratio with competing risk, we predicted that we needed 325 subjects to obtain 91 occurrences of the outcome (CD4 count<350 cells/μl or death) for calculating a sub-hazard ratio of 2 (SHR = 2.0), as significant, with 95% confidence intervals.

Validity of the proportional hazards assumption was tested by including time-dependent covariates in the model, namely the last time seen at a demographic surveillance visit and the last time seen in a clinic. To account for the effect of missing follow-up clinical data (such as ART initiation and CD4 counts), we assessed differences in outcome in those found in clinics compared to those not found in clinics. We pre-specified that we would keep a variable for not being found in a clinic in all models if significant. Finally, a sensitivity analysis was done to assess for bias due to loss-to-follow-up by considering all lost-to-follow-ups as reaching the composite endpoint. Another sensitivity analysis was done to assess for bias due to our definition of *Schistosoma* spp. infection at time of HIV-1 seroconversion. For this sensitivity analysis, we assessed the effect of defining *Schistosoma* spp. infection as having the pre-seroconversion DBS positive for *Schistosoma* spp., instead of requiring both DBS to be positive.

Data were entered into Microsoft Excel and all analyses were performed in STATA 14.1 (College Station, TX, USA). When exact dates were not available, dates were approximated to the 15^th^ of the month if only the month was known or to the 1^st^ of July if only the year was known. All results were expressed with 95% confidence intervals (CIs) and statistical significance was set at P < 0.05 (two-tailed).

### Ethical considerations

Ethical approval for retrospective and prospective analysis of these data was obtained from Bugando Medical Centre in Mwanza (BREC/001/04/2011), the National Institute for Medical Research in Dar es Salaam (NIMR/HQ/R.8a/Vol.IX/2446), and Weill Cornell Medicine in New York (1108011883). Study participants provided consent at the time of enrollment into the cohort study in accordance with the approved procedures of the TAZAMA project, which included consent for future testing of dried blood spot samples [16–17].

## Results

Between September 2006 and February 2016, 172 adults aged 18 years and above HIV-1-seroconverted within the TAZAMA sero-surveys and had stored pre- and post-seroconversion samples available for testing for *Schistosoma* infection. A total of 63/172 (36.6%) seroconverters had a pre-HIV-seroconversion positive test for *Schistosoma*. 43/172 (25.0%) had both samples positive for *Schistosoma*. These 172 HIV-1 seroconverters were followed for a median of 3.4 [2.3–5.4] years from the time of seroconversion to censoring or completion of the study. 98 (57.0%) were found in 16 of the 20 HIV clinics visited and the remaining 74 were found in the demographic surveillance data. 82(85.4%) were known to have initiated ART by March 2017. The baseline characteristics of all seroconverters are presented in Table 1.

**Table 1:** Demographics of the TAZAMA HIV-1 seroconverters, by schistosome infection status at time of HIV-1 seroconversion.

Variable		Schistosome infected N = 43	Schistosome uninfected N = 129	p-value
Female		26/43 (60.5%)	90/129 (69.8%)	0.26
Age in years at HIV-1 seroconversion (Median-IQR)		34[27–47]	35[26,43]	0.79
Smoking		5/36 (13.9%)	12/109 (11.0%)	0.64
Alcohol consumption	Never	30/36 (83.3%)	96/109 (88.1%)	0.48
	Less than once a month	2/36 (5.6%)	4/109 (3.7%)	
	1–3 days per month	1/36 (2.9%)	5/109 (4.6%)	
	1–4 days per week	3/36 (8.3%)	2/109 (1.8%)	
	5–6 days per week	0/36 (0.0%)	1/109 (0.9%)	
	Every day	0/36 (0.0%)	1/109 (0.9%)	
Reported sexually transmitted infection		23/40 (57.5%)	75/126 (59.5%)	0.82
Reported hypertension		1/31 (3.2%)	4/84 (4.8%)	0.72
Tuberculosis positive		2/21 (9.5%)	3/73 (4.1%)	0.33
More than 7 years of education		27/43 (62.8%)	80/129 (62.0%)	0.93
Found in an HIV clinic		23/43 (53.5%)	75/129 (58.1%)	0.59
CD4 < 350 cells/uL		6/23 (26.1%)	33/75 (44.0%)	0.12
Reported death (from demographic surveillance system or clinic)		2/43 (4.7%)	9/129 (7.0%)	0.59
Initiated antiretroviral treatment		21/22 (95.5%)	61/73 (83.6%)	0.13

There were 42 occurrences of the outcome (defined as either reaching CD4 count <350cells/μL or death) in 636.4 person-years of follow-up (4 occurrences in 142.2 person-years for schistosome positive seroconverters and 38 occurrences in 494.2 person-years for schistosome negative seroconverters). 50 adults had initiated ART before reaching a CD4 count <350 cells/μL and therefore had experienced the “competing risk” (defined as starting antiretroviral treatment before reaching the outcome). 35 of the 39 CD4 counts <350 cells/μl occurred before the person had initiated ART and 7 of the 9 deaths happened in adults that had not reached a CD4 count<350 cells/μL and had not started ART, yielding the total of 42 occurrences of the outcome that occurred prior to ART initiation. Schistosome positive seroconverters had experienced the competing risk 33 times while schistosome negative seroconverters had experienced the competing risk 17 times. The differences in the outcome and competing risks are presented in Table 2.

**Table 2:** Results of the univariable competing risk regression based on sub-distribution hazard ratios.

Variable		Person-time (in years)	Number of occurrences of the outcome	Number of competing events	Sub-Hazard Ratio [95%CI]	p-value
*Schistosoma* spp. (ref = CAA negative at HIV-1 seroconversion)	Negative	494.2	38	33	0.31 [0.12,0.84]	0.021
	Positive	142.2	4	17		
Before/After last seen at a demographic surveillance visit (ref = before)	Before	602.4	31	32	4.14 [1.86,9.24]	0.001
	After	34.0	11	18		
Before/After last seen at a clinic (ref = before)	Before	328.2	35	50	0.31 [0.14,0.71]	0.005
	After	308.2	7	0		
Attending clinic (being located in a regional clinic)	No	282.9	5	0	4.09 [1.60,10.43]	0.003
	Yes	353.5	37	50		
Sex	Male	212.9	16	11	0.74 [0.40,1.36]	0.33
	Female	423.5	26	39		
Age (in years)	---	---	---	---	1.03 [1.01,1.05]	0.004
CAA before HIV-seroconversion (in pg/mL)	---	---	---	---	1.00 [0.99–1.00]	0.97

**Fig 1:**
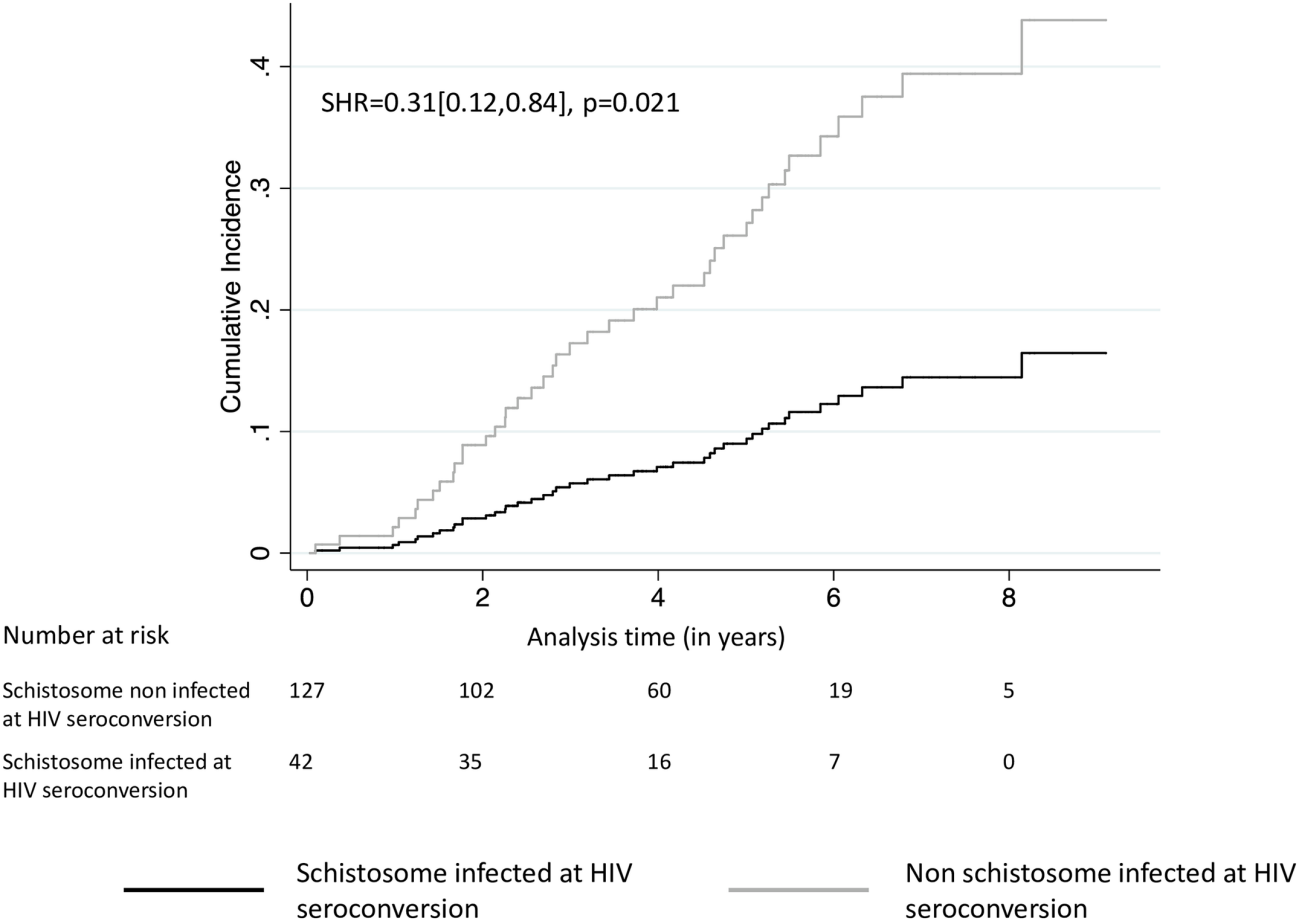
Cumulative incidence function of the composite outcome CD4^+^ T-cell counts <350 cells/μL and/or death, controlling for ART initiation, by schistosome infection status at time of HIV-1 seroconversion. **Time in years**. *The curve represents the cumulative incidence of the composite endpoint while controlling for the competing risk*.

Fig 1 (Cumulative Incidence Function) illustrates endpoint differences between the two schistosome infection groups. The overall outcome incidence was significantly lower in HIV-1 seroconverters infected with *Schistosoma* spp. compared to those non infected with *Schistosoma* spp. (Subdistribution Hazard Ratio (SHR) = 0.31 [0.12,0.84], p = 0.021).

Attending a clinic was significantly associated with a higher rate of the outcome (SHR = 4.09[1.60,10.43], p = 0.003) and all regressions included this variable to account for the effect of missing follow-up clinical data. The rate of the outcome also differed significantly before and after the last demographic surveillance visit and before and after the last clinic visit at which the seroconverters were seen (SHR = 4.14 [1.86, 9.24], p = 0.001 and 0.31[0.14,0.71], p = 0.005, respectively, by univariable analysis). The results of the univariable analyses are presented in Table 2.

After stepwise analysis, the final multivariable model included schistosome infection status, CAA value pre-HIV-seroconversion, age and time of demographic surveillance visit. The impact of schistosome infection on time to outcome was still statistically significant and protective: there was an 82% reduction in risk of reaching a CD4 count <350 or death in HIV-1 seroconverters infected with schistosomes compared to those free of schistosomes (SHR = 0.18[0.068,0.50], p = 0.001) at time of seroconversion. The results of the final model are presented in Table 3.

**Table 3:** Results of the multivariable competing risk regression based on sub-distribution hazard ratios, including control for missing observations and time-dependent covariates, with variables selected by stepwise analysis and model goodness of fit tested for (N = 169).

	Sub-Hazard Ratio	95% CI	p-value
*Schistosoma* spp.-infected (ref = CAA negative at HIV-1 seroconversion)	0.18	[0.068,0.50]	0.001
After seen at a demographic surveillance visit	4.77	[1.67,13.6]	0.003
Age at seroconversion (in years)	1.079	[1.042,1.12]	<0.001
CAA before HIV-seroconversion (in pg/mL)	1.00	[1.00,1.00]	0.004

The sensitivity analysis considered all 75 seroconverters who were lost-to-follow-up before 15^th^ March 2017, as having reached the endpoint. The analysis included 116 occurrences of the outcome and showed a SHR for schistosome infection in the same direction but not statistically significant (SHR = 0.63[0.34,1.15], p = 0.133). When running the analysis for the less stringent definition of schistosome infection, with schistosome positivity at HIV-1 seroconversion defined as having a pre-HIV-seroconversion schistosome positive test, we observed similar results (SHR = 0.60[0.30,1.23], p = 0.16 before adjustment and SHR = 0.44[0.20,0.99], p = 0.047 after adjusting for time-dependent variables, age, and CAA before HIV-seroconversion).

## Discussion

Our study demonstrates that people with schistosome infection at the time of HIV-seroconversion develop adverse HIV outcomes more slowly than those without. Ours is the first study, to our knowledge, that used a longitudinal design to examine the impact of schistosome infection on HIV outcome. Because routine screening and treatment for schistosomiasis was not the standard of care during the follow-up period, our testing of banked samples provides a rare window into the long-term effects of schistosome infection on HIV-1 disease progression. Although our prior work found that HIV-1 seroconverters with schistosome co-infection develop higher HIV-1 viral load set-points [3] and would thus be expected to have more rapid HIV-1 disease progression [14,25], our current longitudinal findings suggest more complex interactions between HIV-1 and schistosome co-infections that may be modulated over time.

Even if HIV-1 viral load set-point is indeed higher in those with HIV-1 schistosome co-infection [3], our long-term follow-up raises the question of whether HIV-1 viral load may later become lower in those with schistosome co-infection than in those with HIV-1 alone. Longitudinal studies in macaques have shown nonsignificant trends in this direction [26–27], but human studies have yielded mixed results, some of which differ by helminth species. In support of this concept, several observational studies in patients with chronic HIV-1 infection have demonstrated higher CD4 counts and/or lower HIV-1 viral loads in those with versus those without helminth co-infection [28–29]. Others have reported transitory increases in HIV-1 viral loads following treatment of schistosome infections [28, 30–32] or no difference in CD4 counts [33–34] and plasma viral load [30,34] between patients with HIV-1/schistosome coinfection and HIV-1 alone. A randomized controlled trial found that providing empiric anti-helminth treatment to Kenyan adults with HIV-1 infection did not delay HIV-1 disease progression [35].

One possible explanation for our findings could be a protective effect of host immune responses to schistosomes against HIV-1 progression. Individuals with chronic schistosome infection have increased peripheral blood percentages and absolute numbers of Th17 cells and T regulatory cells as compared to uninfected individuals, particularly when they have a high degree of schistosome-induced tissue pathology [36–41]. In addition, studies in HIV-infected patients suggest that Th17 and T reg cells, as well as their ratio, may play a critical role in determining the speed of HIV/AIDS progression [42–43]. Lower Th17/Treg ratios have been associated with more advanced HIV-1 infection [44], while absolute increases in T reg numbers have been associated with decreased markers of immune activation [44–45], potentially leading to better HIV-1 outcomes. In addition, HIV-1 so-called “elite controllers”, who maintain very low HIV-1 viral loads and high CD4 counts without antiretroviral therapy, have been found to have higher baseline numbers of Th17 cells than other HIV-1-infected individuals, possibly because more Th17 cells could prevent microbial translocation and thereby decrease immune hyperactivation [43–44]. Taken together, this body of evidence suggests that one possible immunologic mechanism to explain our study’s findings could be the induction of Th17 and T reg cells by chronic schistosome infection, leading to delayed HIV-1 disease progression.

Age did not modify HIV-1 disease progression, contrary to what previous studies had shown [46–47], likely due to homogeneity in age between the two groups. It is surprising that sex was not significantly associated with HIV/AIDS progression given that studies demonstrate higher HIV-1 viral load set-points in men than in women [48–50]. It is possible that other variables were so strongly associated with the outcome that the sex effect became relatively inconsequential, that sex differences in linkage to care were small in our study, or that the complexities of interactions between host sex and schistosome infection [51] make detection of a simple relationship difficult.

Our results are to be interpreted in light of some limitations. The sero-surveys were conducted every 3 years, which only allowed us to approximate seroconversion dates and to assume that schistosome infection status at seroconversion was correlated to the infection status at the last HIV-1 negative sero-survey and first HIV-1 positive sero-survey. We were also unable to test for viral loads, or additional immunologic markers that might provide insight into the reasons for our observations, due to insufficient quantity of blood in DBS. We also assumed that people not found at a clinic did not go to an HIV clinic, which is not necessarily true, especially for people who moved from the study area shortly after seroconversion. If individuals with schistosome infection tended to be more mobile or to attend clinics outside of our catchment area, fewer of them would have reached the CD4 endpoint and some deaths could have been missed. Finally, we identified ART use as the most important competing risk that could have impacted CD4 counts and mortality, but it is possible that we did not account for other important competing risks. The sensitivity analysis considering lost-to-follow-ups as dead did not yield statistically significant results, suggesting that the analysis is sensitive to differential loss to follow up. However our assumption that all lost-to-follow-ups died is quite extreme, and the fact that the SHR yields a result in the same direction, with a p-value of 0.13, reinforces the idea that schistosome infection is associated with decreased incidence of negative HIV/AIDS outcomes.

In conclusion, our study suggests that schistosome infection at the time of HIV-1 acquisition may delay HIV-1 disease progression. Complementary findings from a variety of other studies of HIV-1/helminth co-infections strengthen the likelihood that this result is not spurious. Plausible mechanisms by which schistosome infection could delay HIV-1 disease progression include induction of Th17 or T reg cells or disrupting the Th17/Treg ratio. This work highlights the need for additional studies to examine these immunological interactions between the two pathogens on a longer-term scale.
